# The Role of Interleukine-10 and Interferon-γ as Potential Markers of the Evolution of African Swine Fever Virus Infection in Wild Boar

**DOI:** 10.3390/pathogens10060757

**Published:** 2021-06-15

**Authors:** Sandra Barroso-Arévalo, Jose A. Barasona, Estefanía Cadenas-Fernández, Jose M. Sánchez-Vizcaíno

**Affiliations:** 1 VISAVET Health Surveillance Center, Complutense University of Madrid, 28040 Madrid, Spain; jbarason@ucm.es (J.A.B.); estefaca@ucm.es (E.C.-F.); jmvizcaino@ucm.es (J.M.S.-V.); 2 Department of Animal Health, Faculty of Veterinary, Complutense University of Madrid, 28040 Madrid, Spain

**Keywords:** ASF, cytokines, IL-10, IFN-γ, wild boar, vaccine candidate

## Abstract

African swine fever virus (ASFv) is one of the most challenging pathogens to affect both domestic and wild pigs. The disease has now spread to Europe and Asia, causing great damage to the pig industry. Although no commercial vaccine with which to control the disease is, as yet, available, some potential vaccine candidates have shown good results in terms of protection. However, little is known about the host immune mechanisms underlying that protection, especially in wild boar, which is the main reservoir of the disease in Europe. Here, we study the role played by two cytokines (IL-10 and IFN-γ) in wild boar orally inoculated with the attenuated vaccine candidate Lv17/WB/Rie1 and challenged with a virulent ASFv genotype II isolate. A group of naïve wild boar challenged with the latter isolate was also established as a control group. Our results showed that both cytokines play a key role in protecting the host against the challenge virus. While high levels of IL-10 in serum may trigger an immune system malfunctioning in challenged animals, the provision of stable levels of this cytokine over time may help to control the disease. This, together with high and timely induction of IFN-γ by the vaccine candidate, could help protect animals from fatal outcomes. Further studies should be conducted in order to support these preliminary results and confirm the role of these two cytokines as potential markers of the evolution of ASFV infection.

## 1. Introduction

African swine fever (ASF) is a hemorrhagic disease that exclusively affects animals belonging to *Suidae* family. It is caused by a DNA virus that is the only member of the *Asfarviridae* family [1]. After its eradication from the Iberian Peninsula in the 1990s, the disease persisted solely in most sub-Saharan countries in Africa, with the exception of the island of Sardinia. However, from 2007 onward, ASF spread to Eastern Europe and later into Western Europe and Asia. Since the initial outbreak in Georgia, ASF has affected both domestic pigs and wild boar (*Sus scrofa*) in most of Eurasia [2]. The expanding wild boar populations, in which the disease has established self-sustaining cycles, contribute to the maintenance of the disease and its recent spread in Europe [3] and Asia [4].

A large number of isolates of the ASF virus (ASFV) have been described [5,6], with diverse levels of virulence. The result of the infection differs significantly not only among different strains but also within hosts owing to both species-specific and virus factors [7]. In Africa, wild African suids are tolerant to ASFV infection and may, therefore, act as reservoirs for the disease [8]. The infection may, in contrast, have devastating consequences in other suid hosts, with lethality rates ranging from 30% in the case of moderate virulent isolates to 100% in that of highly virulent isolates [9]. This reflects the fact that certain factors of the hosts may play a critical role in overcoming the disease. Although descriptions of neutralizing antibodies against the structural proteins p30, p54, and p72 have been provided [10], the further reproducibility of these results has not been possible [11]. It has, therefore, been postulated that cellular-mediated mechanisms may contribute to the outcome of the infection [9,12].

The virulence of the isolate may also be essential to the development of an effective immune response. This is particularly relevant in the case of effective vaccine development since most ASFV vaccine candidates are live attenuated strains, examples of which are the naturally attenuated ASFV genotype I isolates NH/P68 and OURT88/3 [13,14,15]. Another example is the weakly virulent, non-hemadsorbing ASFV strain Lv17/WB/Rie1, which has proven to provide over 92% protection against a challenge with a virulent ASFV genotype II isolate Armenia07 (hereafter, Arm07) in both domestic pigs and wild boar [16,17], the latter of which is the main reservoir for the disease in Europe [18,19,20]. However, little is known about what makes one isolate less virulent than others or which host factors are involved in an effective immune response, which is of great importance in vaccine development. Although safety concerns have limited the implementation of live attenuated vaccines in the field, they are the best candidates at present and are of great use in regards to attaining a better understanding of the role that both specific antibodies and T cells play in ASFV protection [21].

The primary targets of ASFV are macrophages and monocytes. These cells play a critical role in the immune system, as they can initiate the immune response by secreting cytokines, which have antigens, and clearing pathogens by means of phagocytosis [22]. ASFV principally replicates in the cytoplasm of these host cells, where it encodes the enzymes and factors required for genome replication and transcription, but the virus additionally provides considerable coding capacity to genes that help the evasion of host’s defenses [23]. In this respect, ASFV can interfere in the expression of a large number of immunomodulatory genes, such as those in charge of producing pro-inflammatory cytokines [24,25]. Recent studies have proposed that virulent ASFV isolates have developed several mechanisms with which to mitigate macrophage responses, whereas the expression of key cytokines and chemokines from macrophages in response to attenuated ASFV strains could promote the induction of innate immune responses and stimulate the initiation of an effective adaptive immune response [26]. It has also been suggested that ASFV pathogenesis is primarily mediated by host cytokines produced by infected monocytes and macrophages [27,28,29]. Furthermore, several studies have demonstrated that multiple ASFV genes are involved in host immune evasion [7,30]. Vaccine development should, therefore, target this species and explore the host immune response triggered by these vaccine candidates.

Sánchez-Cordón et al. [31] postulated that the early induction of interferon-gamma (IFN-γ) and interleukin 10 (IL-10) in vaccinated pigs might be critical regarding controlling the initial virus replication that triggers the immunological mechanisms, which may encourage their survival after a challenge [31]. IFN-γ plays a key role in viral infections through the activation of NK cells and macrophages and by increasing their phagocytic capacity [32]. For instance, the deletion of genes related to IFN inhibitory proteins from virulent isolates has resulted in virus attenuation [33,34,35,36]. It is, however, still unclear whether the induction of type II IFN can be considered as a reliable indicator of protection in vaccinated animals [36,37,38]. In the case of IL-10, recent studies have postulated that this cytokine might play a key role in the ASFV strategy in regards to interfering in the generation of a specific immune response by controlling antiviral IFNs levels and a cell-mediated immune response [38]. Previous experimental vaccine trials have also suggested that IL-10 might help control the first steps of viral replication and mitigate the damaging costs of an intensified inflammatory response that is characteristic of acute ASFV infections [31]. However, most experiments have been conducted on pigs, and little is, therefore, known about the role of this cytokine in ASFV-infected wild boar.

In this paper, we study the immune response by analyzing and quantifying the presence of IFN-γ and IL-10 in wild boar inoculated with a potential vaccine candidate (the attenuated Lv17/WB/Rie1 isolate) and subsequently challenged with the virulent Arm07 isolate. A control group formed of naïve (not vaccinated) animals was established and challenged with the virulent Arm07 isolate. The levels of both cytokines were evaluated at different time points in order to elucidate the role played by these cytokines during ASFV infection and the mechanisms underlying the protection capacity of this vaccine candidate in wild boar.

## 2. Results

### 2.1. Clinical Signs, ASFV DNA Detection, and Antibody Response of Wild Boar from the Vaccinated Group

Animals from the vaccinated group did not show any significant clinical signs during the 30 days prior to the challenge (vaccination period), apart from a slight increase in body temperature to 40.1–40.8 °C in eight of the eleven animals, which lasted a mean of 3.5 days, starting from 4 to 24 days post-inoculation. Virus genome levels were evaluated in blood samples taken throughout the experiment by means of real-time PCR, which attained weakly positive results (Ct = 33.02 ± 4.07) during the vaccination period (Supplementary Materials Scheme S1). Antibodies were detected from 15 ± 2 days after Lv17/WB/Rie1 inoculation based on ELISA and IPT tests, and titers remained high throughout the experiment, including the post-challenge period.

According to the exclusions employed to select the animals in this group, all the animals survived after the challenge, and no ASF-compatible clinical signs were detected. Five of the eleven animals sporadically showed weak viremia peaks (Ct = 35.78 ± 2.20). No pathological findings consistent with ASF were found in the animals during the post-mortem evaluation.

### 2.2. Clinical Signs, ASFV DNA Detection, and Antibody Response of Wild Boar from the Control Group

All the animals died or were euthanized on the basis of humane endpoints, at 14 ± 1 day post-infection. The main clinical signs in these animals were high fever, partial lethargy, and slight anorexia. Viremia was detected by employing qPCR and started at 11 ± 2 days post-contact (Ct = 28.67 ± 4.39) (Supplementary Materials Scheme S1). Only three of the eleven animals tested positive for anti-ASFV antibodies at 11, 13, and 14 days post-contact, respectively. Pathological findings consistent with ASF were found in all the animals during the post-mortem evaluation.

### 2.3. Survival Analysis

**Figure 1:**
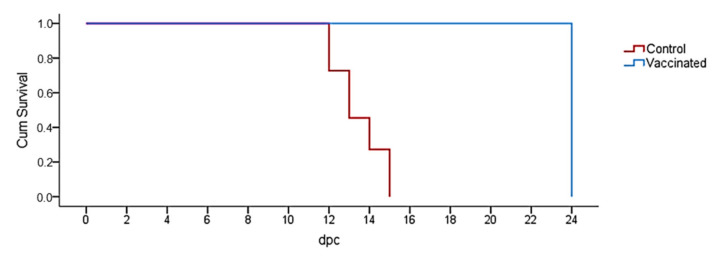
Overall survival after challenge with the virulent isolate Armenia07 of African swine fever virus. Red color represents animals from Control group; blue color represents animals from Vaccinated group.

Vaccinated animals survived the whole experiment period after challenge, while animals from control group died or were euthanized at 14 ± 1 day post-infection, as described above. A Kaplan–Meier plot is shown in Figure 1 (*p* < 0.001).

### 2.4. Cytokine Levels in Serum

The role played by cytokines in the regulation of the immune response was evaluated. The levels of IL-10 and IFN-γ in serum were measured at different points for both groups.

**Figure 2:**
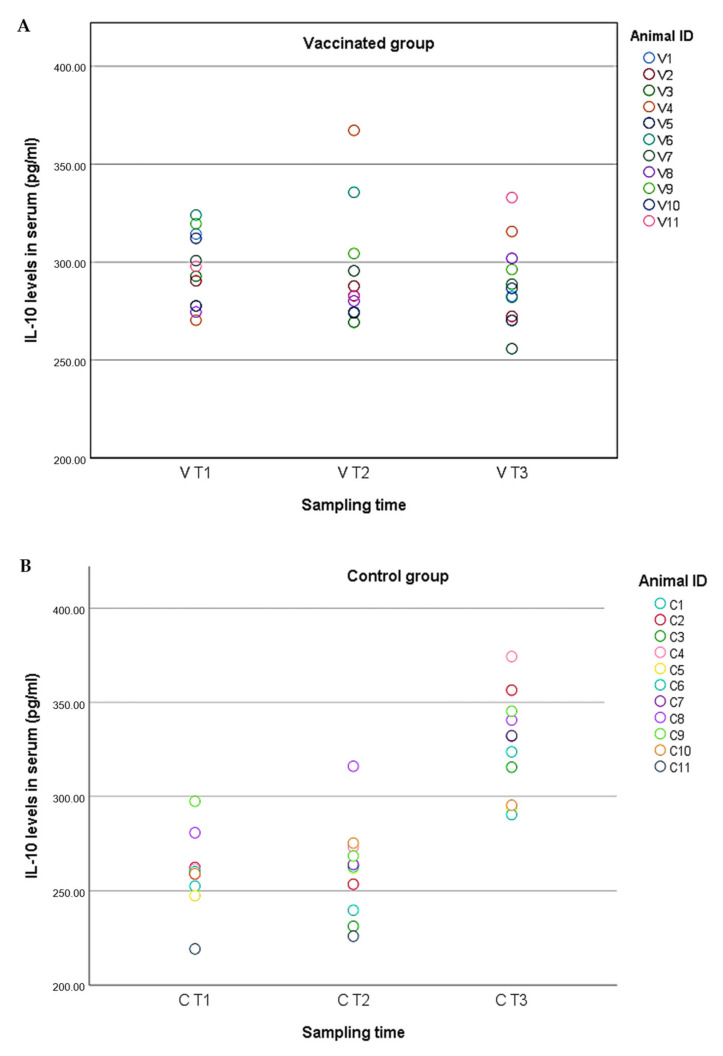
(**A**) Interleukin 10 (IL-10) levels in serum in vaccinated wild boar (*n* = 11, V1–V11) based on the sampling time (T1: day 0; T2: the day animals started producing antibodies; T3: after challenge). Animal ID: vaccinated, V, from 1 to 11. (**B**) Interleukin 10 (IL-10) levels in serum in control group (*n* = 11, C1–C11) based on the sampling time (T1: day 0; T2: 6 days before the challenge; T3: after challenge). Animal ID: control, C, from 1 to 11.

**Figure 3:**
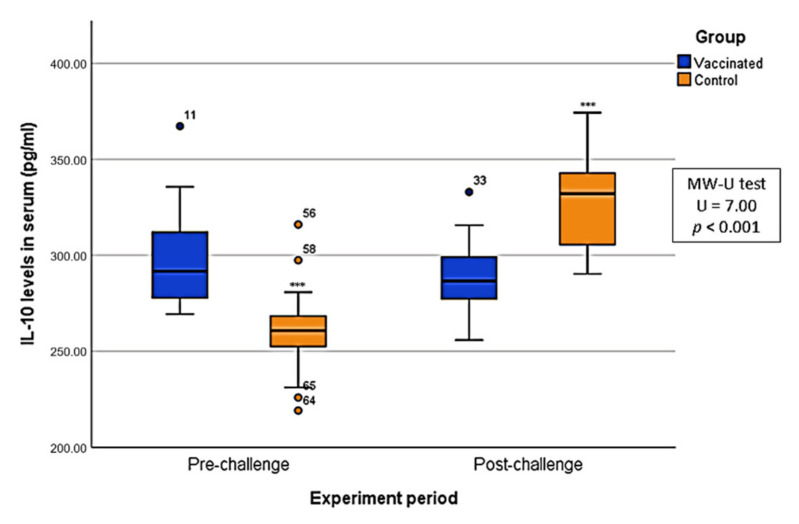
Box plot comparing interleukin 10 (IL-10) levels in serum in both vaccinated and control groups based on the periods of the experiment (pre and post-challenge with the virulent Arm07 isolate). *p*-Values: = 0.1; *** = *p* < 0.001.

With regard to the animals from the vaccinated group, the IL-10 levels remained relatively constant during the experiment, with values ranging from 255.81 to 367.24 pg/mL and an average of 297.68 pg/mL in V T1, 295.86 pg/mL in V T2, and 289.55 in V T3 (Figure 2A). There were no significant differences among the three different periods (KW; *p* > 0.05) or between pre- and post-challenge (MW-U; *p* > 0.05). In the case of the animals from the control group, however, the IL-10 levels were less constant during the experiment, with values ranging from 219.21 to 374.30, and an average of 259.91 pg/mL in C T1, 261.07 pg/mL in C T2, and 327.22 pg/mL in C T3 (Figure 2B). The IL-10 levels in these animals pre-challenge were significantly lower than in post-challenge (MW-U; U = 7.00; *p* < 0.001) (Figure 3).

**Figure 4:**
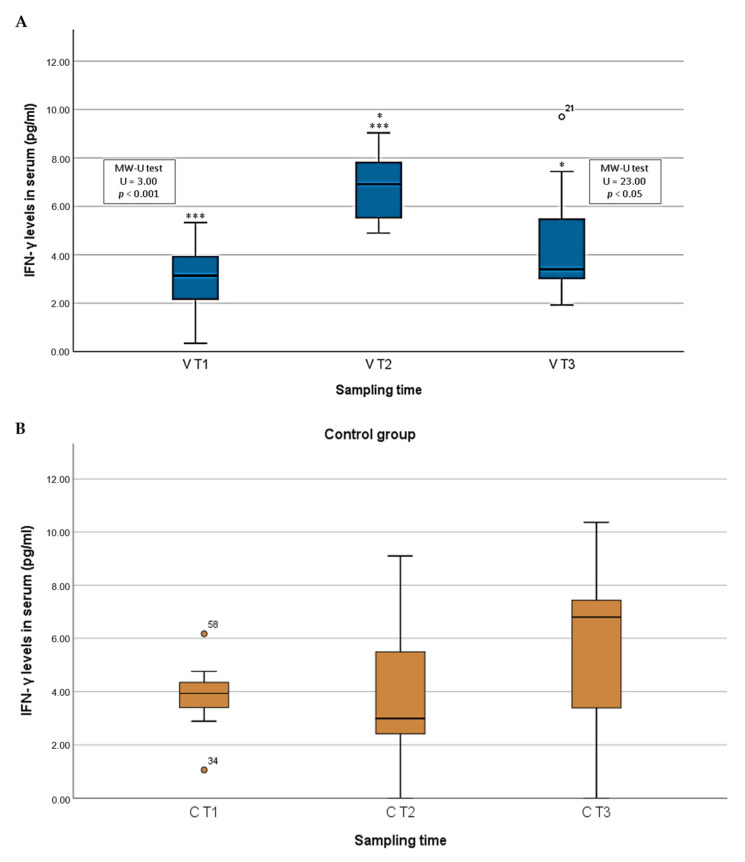
(**A**) Box plot comparing interferon-gamma (IFN-γ) levels in wild boar from vaccinated group inoculated with the attenuated Lv17/WB/Rie1 isolate (blue color) and then challenged with the virulent Arm07 strain at different time points (T1: day 0; T2: the day animals started producing antibodies; T3: after challenge). (**B**) Box plot comparing interferon-gamma (IFN-γ) levels in wild boar from control group inoculated with the virulent Arm07 strain (orange color) at different time points (T1: day 0; T2: 6 days before challenge; T3: after challenge). *p*-Values: = 0.1; * = *p* < 0.05; *** = *p* < 0.001.

IFN-γ levels fluctuated within groups. The IFN-γ levels in the vaccinated animals ranged from 0.34 to 14.59, with an average of 2.99 pg/mL in V T1, 7.32 pg/mL in V T2, and 4.47 in V T3. Significant differences were detected among the different sampling points. IFN-γ increased at the moment the animals started producing antibodies when compared to the level observed on day 0 (MW-U, U = 3.00, *p* < 0.001). After the challenge, the level of IFN-γ then decreased when compared to the level observed in the onset of antibody response (MW-U, U = 23.00, *p* = 0.01), and there was no significant difference from the level observed on day 0 (MW-U, *p* > 0.05), as is shown in Figure 4A.

The IFN-γ levels in the animals from control group ranged from 0 to 10.37, with an average of 3.80 pg/mL in C T1, 4.01 pg/mL in C T2, and 5.53 in C T3. No significant differences among the three sampling points were detected in the IFN-γ levels in serum (KW; *p* > 0.05). Although no significant differences were found, a slight increase in IFN-γ levels was detected after the challenge (Figure 4B).

## 3. Discussion

The results of this study can be used as a starting point to decipher part of the immune response in wild boar that were orally inoculated with a potential vaccine candidate (the attenuated isolate Lv17/WB/Rie1) and that subsequently survived the challenge with a virulent Arm07 isolate. The analysis and quantification of the presence of IFN-γ and IL-10, which have previously been shown to modulate ASFV infection in domestic pigs, were different when compared to those carried out for a control group of naive (unvaccinated) wild boar challenged with the same virulent Arm07 isolate. Moreover, the levels of both cytokines quantitatively varied over the immunization period and ASFV infection. These results can be employed as a basis on which to unravel the mechanisms underlying the protective capacity of this vaccine candidate in wild boar.

Despite the fact that this potential vaccine candidate against ASFV has provided successful results in terms of protection in both pigs and wild boar [16,17], there is a lack of knowledge regarding the mechanisms underlying its safeguarding capacity [9,21]. Although humoral response would appear to be required in order to control the disease, classical virus-neutralizing antibodies have not been identified in the presence of ASFV infection, in a similar way that anti-virus antibodies often coexist with viremia without protecting the host [39]. However, it cannot be dismissed that antibodies play an active role against ASFv infection. Alternative cellular-based immune mechanisms may, therefore, be involved in promoting an effective response to the infection [40], as is suggested by our results, in which IL-10 and IFN-γ may be used as potential markers of immune system response against ASFV infection. Furthermore, host immune and/or concurrent co-pathogen infection status appear to affect the outcome of the disease. Although some studies on these topics have been carried out in pigs, little is known about immune host factors in the wild boar, which is the main reservoir of ASFV in the current epidemic scenario [18,19]. This is, to the best of our knowledge, the first study to evaluate a cytokine-based response in the presence of ASFV infection in wild boar.

Although the efficacy of the vaccine candidate Lv17/WB/Rie1 has been proven, there are still major concerns that need to be tackled. These include a safety evaluation, high biocontainment requirements for the production of the attenuated virus, the availability of suitable cell lines, and the optimization of culture conditions for vaccine virus production [9,41]. It will also be necessary to carry out an in-depth analysis with which to evaluate the interaction between the virus and the host immune system.

IL-10 levels varied between groups. Vaccinated animals showed a slight peak in IL-10 production after vaccine inoculation, but this was not statistically significant and levels of this cytokine remained stable during the whole experiment, including the period after the challenge with the virulent isolate. In contrast, significant differences in IL-10 levels within the three sampling times were found in the non-protected control group. These animals showed an increase in the production of this cytokine after the challenge, coinciding with the peak of fever, symptoms, and death. IL-10 is an anti-inflammatory cytokine produced by regulatory T cells, which fulfills an essential function in the regulation of those cells [42]. IL-10 is also involved in the inhibition of NK cells and the adaptive cellular immune response, the down-regulation of major histocompatibility complex class II (MHC-II) expression, and the suppression of pro-inflammatory cytokines involved in several immune functions, such as the growth of different cell types, the activation of macrophages, antibody production, and chemotactic mechanisms [43]. The central role played by IL-10 as an immunomodulatory cytokine has led several studies to report that some viruses may encourage the induction of IL-10 by T regulatory cells to persist in the host by decreasing the level of IFN-γ production and cytotoxic activity [44]. However, the increase in IL-10 levels post-challenge in the control group was not accompanied by a decrease in IFN-γ levels in serum as might have been expected, but may reflect a disproportionate immune response against the virus in those animals. Since IL-10 has the ability to suppress innate and adaptive responses [43], a high level of this cytokine might favor viral pathogenesis by promoting immune system failure, as has been reported by other authors [15,45]. The vaccinated animals, however, had constant and lower levels of IL-10 during the post-challenge period. The attenuated isolate might, therefore, induce a more controlled IL-10 production, which would enhance a purely anti-inflammatory response with which to counter ASFV without devastating consequences for the immune system.

These results contrast with a recent in vitro study in which pathways involved in IL-10 production were down-regulated in the presence of ASFV [29]. The authors of the study in question suggested that the down-regulation of IL-10 expression in macrophages could have a significant enhancing effect of those pro-inflammatory cytokines on the pathogenesis of ASF. However, our results are similar to those of another in vivo study in which high levels of IL-10 after challenge were postulated to be involved in a fatal outcome [15,45]. Although in vitro studies are necessary to establish the basis of any knowledge, in vivo and field studies must be performed to confirm those findings in this host–pathogen system.

IFN-γ levels in serum were also evaluated in both the vaccinated and control animals, with different patterns being attained for each group. In the case of the vaccinated-protected animals, IFN-γ levels started at a low degree. After immunization with the Lv7/WB/Rie1 vaccine candidate, the animals began to produce specific antibodies against ASFV, which coincided with a significant increase in IFN-γ levels. After challenge with the virulent Arm07 isolate, cytokine levels declined to low values, but they were slightly higher than the initial ones. There were, however, no significant differences in the IFN-γ levels of the animals from the control group, although a small increase was detected after the challenge. The IFN system regulates innate and adaptive immunity to viral infection. Viral invasion directly triggers the induction of type I IFN (the IFN-γ), which is crucial to the immediate cellular response to viral infection. The immunomodulatory activities of this cytokine include the induction of antiviral enzymes such as PKR, the coordination of the transition from innate to adaptive immunity, or the control of the production of other cytokines, among others. Several studies have reported the importance of IFN-γ during ASFVv infection [31,35,38]. Some of the strategies performed by viruses in order to evade the host immune response are the inhibition of IFN-γ production, the inhibition of IFN-mediated signaling pathways, and the blocking of the action of IFN-induced enzymes with antiviral activity [30]. In the specific case of ASFV, protective response against the virus has been reported to be mediated by subsets of NK cells and CD8 + T-cells [14,46]. The peak of this cytokine in vaccinated-protected animals coinciding with the start of the antibody production may, therefore, reflect an adequate immune response. The combination of a strong humoral response and high levels of IFN-γ might have protected the vaccinated animals from the virulent isolate. These results coincide with those of previous studies, in which elevated IFN-γ levels have been associated with specific immune responses [14,47,48]. For instance, it has been reported that the deletion of IFN-γ inhibitor genes DP148R, MGF360, and 530/505 genes from ASFV Benin97/1 isolate induced protective immune responses against a challenge [35], thus showing the importance of this cytokine in regards to triggering an effective immune response against the virus. In another study, cross-protection induced by the OURT88/3 isolate against a challenge with virulent isolates from different genotypes was correlated with the ability of those isolates to specifically stimulate IFNγ-producing lymphocytes from immunized pigs [47]. However, other authors have suggested that high IFN-γ levels in serum in the last stages of ASFV might be the result of an adverse pathological condition without protective functions [15]. Our findings sit somewhere between these two claims, since we also observed a slight (but not significant) increase in IFN-γ levels in serum in non-protected wild boar after the challenge. However, further research should be conducted in order to better understand the complex relationship between antiviral host mechanisms and viral strategies for antiviral evasion.

## 4. Materials and Methods

### 4.1. Animals

Experiments were performed under biosafety level 3 conditions in the VISAVET center at the University Complutense of Madrid, Spain. Two experimental groups were established for this study, which were derived from two previously described independent experiments [16,48]. The first consisted of vaccinated animals who survived the challenge with a virulent isolate as a model of protected animals (vaccinated group). The second consisted of a group of animals that did not survive the infection with a virulent isolate as a model of non-protected animals (control group). Each group consisted of 11 female 3–4-month-old wild boar piglets weighing 10–15 kg, which were obtained from a commercial wild boar farm in Extremadura, Spain. The animals were acclimated for 2 weeks before the experiment began. During the acclimatization phase, the wild boar received metaphylactic treatment with oxytetracycline dihydrate (Alamycin LA 300, Norbrook Laboratories, Newry, UK) and ivermectin (Ivomec S, Merial GmbH) in order to eliminate parasites and to control any unapparent bacterial infections. These wild boar piglets had not been vaccinated, and tested negative for the following main porcine pathogens in the region: Aujeszky disease virus, *Mycobacterium bovis*, *Mycoplasma hyopneumoniae*, and porcine circovirus type 2. Animal care and procedures were performed in accordance with the guidelines for Good Clinical Practice (GCP), following European, national, and regional regulations and under the supervision and approval of the Ethics Committee of the Comunidad de Madrid (reference PROEX 124/18 and 004/18).

### 4.2. Study Design

In the vaccinated group, eight of the eleven wild boar were orally inoculated with 10^4^ TCID_50_ (since this amount of virus causes cytopathic effects in 50% of infected cultures) of the attenuated Lv17/WB/Rie1 isolate as a potential vaccine candidate, whereas the other three wild boar were naïve and left in direct contact with these orally inoculated animals (*n* = 11). There were no significant differences among these last three animals in terms of antibody response and protection against the challenge with respect to the orally vaccinated animals [16], and we, therefore, considered them to be from the same group as a model of protected animals. Thirty days after inoculation and contact (vaccination period), all these animals were exposed to four animals that had been intramuscularly inoculated with 10 HAD_50_ (since this amount of virus causes hemadsorption in 50% of infected cultures) of virulent ASFV Armenia07 isolate (Arm07) as a shedder-pig challenge exposure model. For this retrospective study, we included all the animals that survived the challenge, and they were maintained for 24 more days, as described in [16].

The second group was a control group, which consisted of eleven naïve wild boar that were exposed to two animals that had been intramuscularly inoculated with 10 HAD_50_ of ASFV Arm07 isolate as a shedder-pig challenge exposure model, similar to that which occurs with natural contact infection, as described by Rodríguez-Bertos et al., 2020. None of these animals survived the challenge.

### 4.3. Sampling and Clinical Evaluation

All the animals were individually sampled twice a week. EDTA blood and serum samples were collected from ophthalmic sinus [49] and were processed immediately after collection.

Rectal temperatures and clinical signs were monitored as described in [16,50]. The recorded clinical score was used to define individualized humane endpoints during the experiments. Euthanasia was performed following the procedures of Cadenas-Fernández et al., 2020, if the accumulative clinical score was >18, or if the animals had any of the following severe clinical signs (level 4) for more than two consecutive days: fever, anorexia, recumbence, respiratory or digestive symptoms. Animals that were unacceptably suffering without reaching the endpoint were also euthanized, using veterinary criteria as a basis. Macroscopic lesions were evaluated during necropsies using scores based on a previous standardized protocol [50].

### 4.4. ASFV DNA, Antibody, and Cytokine Detection

DNA was extracted from EDTA blood samples using the High Pure Template Preparation Mix Kit (Roche Diagnostics GmbH, Mannheim, Germany) according to the manufacturer’s instructions. ASFV DNA was detected using real-time PCR (King et al., 2003).

Serum samples were assayed using a commercial competition ELISA kit for the detection of ASFV-specific antibodies against VP72 (INGEZIM PPA3 Compac, Ingenasa, Madrid, Spain) and commercial ELISA kits for the detection of porcine immunoregulatory cytokines (IFN-γ and IL-10, R&D System, Abingdon, UK), according to the manufacturer’s instructions. ELISA results for ASFV-specific antibodies were also confirmed using an indirect immunoperoxidase test (IPT) [51].

Whereas ASFV DNA presence and antibody response were evaluated in each sampling, cytokine detection was performed at three time points of each experiment. In the vaccinated group, cytokines were analyzed on day 0 (VT1: before immunization), when the animals started producing antibodies (VT2: individually selected for each animal; average of 28 days post-exposure) and after the challenge (41 days post-inoculation in the case of the orally vaccinated animals and 52 days post contact in that of the animals immunized by contact with the orally vaccinated animals) with the virulent isolate (VT3). In the control group, cytokines were analyzed on day 0 (CT1), 6 days before the challenge (CT2), and 1–2 days before the animals succumbed to the challenge (CT3).

### 4.5. Data Analysis

Statistical analyses and graphics were performed using SPSS 20 (IBM, Somar, New York, USA) and the Python programming language. A descriptive analysis of ASFV viremia (Ct values) in blood and cytokine levels (pg/mL) in serum was performed to calculate average ranges per sampling time and 95% confidence intervals. Kaplan–Meier curves were created to perform survival analysis. The temporal variation in the cytokine levels between groups and among different sampling times were studied using the Mann–Whitney U Test (MW-U) and the Kruskal–Wallis test (KW), respectively.

## 5. Conclusions

Here, we conclude that IL-10 and IFN-γ play a key role in both effective and ineffective responses against ASFV and that there are different patterns in their expression depending on the type of isolate (attenuated or virulent), which are potential immune markers in the presence of ASFV infection. In this respect, the attenuated isolate tested in this study would appear to induce a more effective immune response in wild boar, with a controlled production of IL-10 and a high and time-adequate induction of IFN-γ, both of which helped control viral replication and pathogenesis when animals were challenged with the virulent isolate. In non-protected animals, however, the challenge virus appeared to trigger a disproportionate increase in IL-10 with no adequate levels of IFN-γ, probably as the result of an ineffective cellular immune response. These two cytokines could, therefore, be used as potential immune markers of the evolution of ASFV infection. This is, to the best of our knowledge, the first study to report the role of these two cytokines in wild boar, which is currently the main reservoir for the disease in Europe. As has been shown in the case of pigs, investigating the immune mechanisms derived from the different vaccine candidates is one of the essential pillars for ASFV vaccine development. Our findings, therefore, contribute to a growing body of literature on the immune response against ASFV and emphasize the importance of understanding the interaction of pathogens and host factors when investigating potential vaccine candidates.

## Data Availability

The data presented in this study are available on request from the corresponding author.

## Supplementary Materials

The following are available online at https://www.mdpi.com/article/10.3390/pathogens10060757/s1, Scheme S1. Real time PCR Ct values, clinical score, and rectal temperature in both Vaccinated and Control groups.
