# Supplementary figures and images for: The Role of Interleukine-10 and Interferon-γ as Potential Markers of the Evolution of African Swine Fever Virus Infection in Wild Boar
Source: Pathogens. 2021 Jun 15;10(6):757. doi: 10.3390/pathogens10060757 (PMC8232672; doi:10.3390/pathogens10060757)

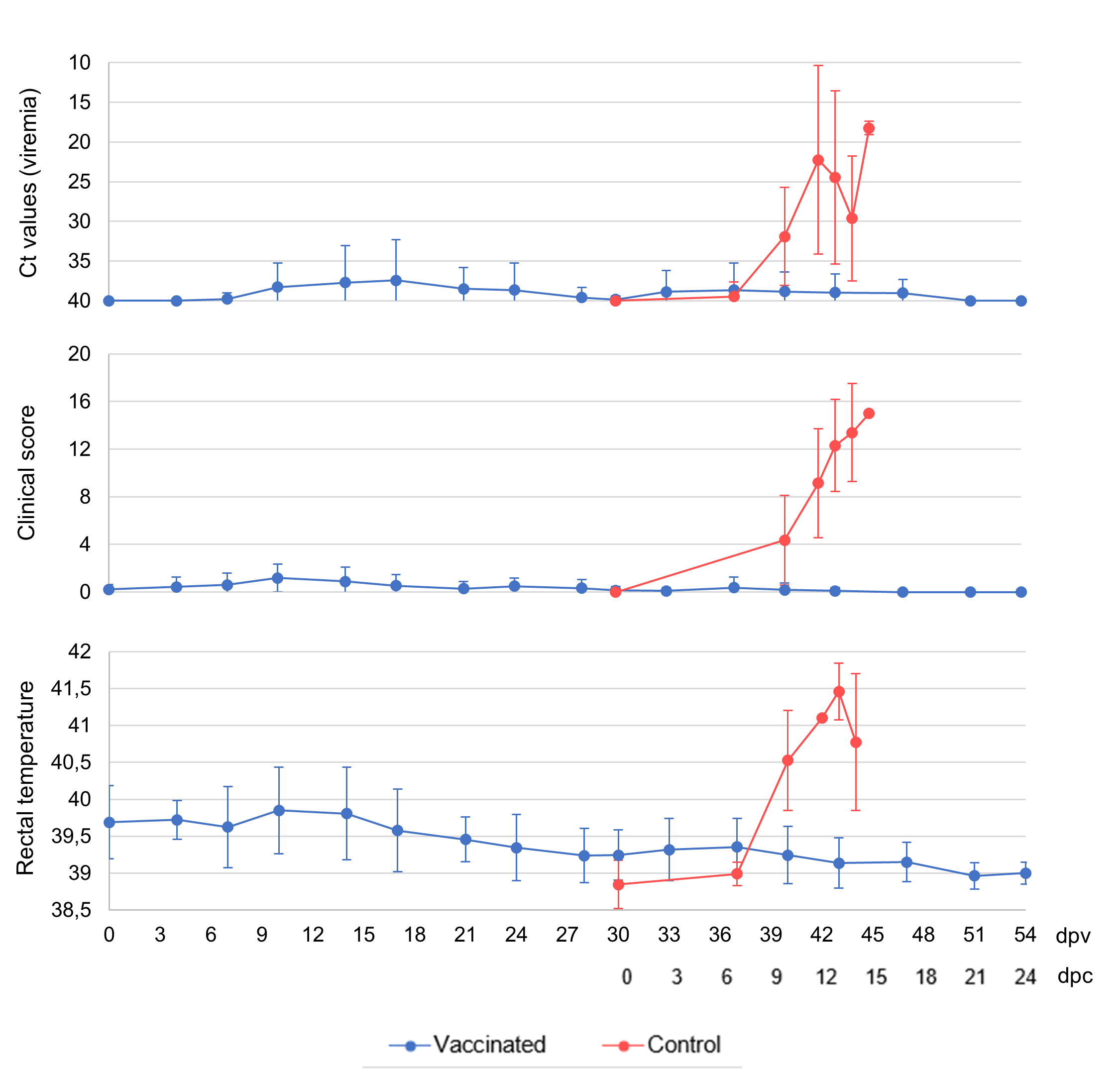

Supplement: Supplementary file 1 [file pathogens-10-00757-s001.zip › pathogens-1244022-supplementary.tif]
